# A VLP-based mRNA vaccine elicits potent humoral and cellular immunity against Oropouche virus

**DOI:** 10.1128/mbio.03653-25

**Published:** 2026-01-12

**Authors:** Yuren Shi, Guangxu Zhang, Siyu Lin, Mengyu Hu, Yuanzhou Wang, Haoyu Ge, Shuai Xia, Qian Wang, Shibo Jiang, Lu Lu

**Affiliations:** 1 Key Laboratory of Medical Molecular Virology (Ministry of Education/National Health Commission/Chinese Academy of Medical Science), Shanghai Institute of Infectious Disease and Biosecurity, School of Basic Medical Sciences, Shanghai Frontiers Science Center of Pathogenic Microbes and Infection, Shanghai Public Health Clinical Center, Fudan University 34748, Shanghai, China; 2 School of Basic Medicine, Dali University 12618, Dali, China; Tsinghua University, Beijing, China

**Keywords:** Oropouche virus (OROV), mRNA vaccine, virus-like particles (VLPs), cross-reactive antibodies

## Abstract

Oropouche virus (OROV) is reemerging in the Americas, along with a growing threat to global public health. Recent outbreaks have witnessed the first reported fatalities, vertical transmissions, and intercontinental importations of OROV, underscoring its expanding risk. Despite this, no vaccines or specific therapeutics are available, and fundamental research on OROV vaccinology and antigenicity remains limited. Here, we show that co-expression of the M polyprotein and nucleocapsid protein (NP) drives the assembly of OROV virus-like particles (VLPs) with high immunogenicity. Using the prototype strain OROV/sloth/Brazil/PA-UG-BeAn19991/1960, we developed an mRNA vaccine, M/N-vac, encoding these VLPs. Immunization with M/N-vac in mice elicited robust OROV-specific IgG and pseudovirus-neutralizing antibodies that cross-reacted with a contemporary circulating strain, hOROV/Brazil/AM-UKY-AM0088/2024. The vaccine also induced a durable, antigen-specific Th1-biased cellular immune response characterized by high-level interferon-gamma secretion. Additionally, we identified a highly conserved potential immunodominant epitope in BALB/c, N2-3, within the nucleocapsid protein. Furthermore, the VLP-encoding mRNA vaccine induced stronger OROV-specific humoral and cellular immune responses than the VLP protein vaccine. *In vivo* results based on immunization with M/N-vac demonstrate VLP-based vaccines to be a promising broad-spectrum strategy against OROV while providing novel insights into their antigenicity and design.

**IMPORTANCE:**

Oropouche virus (OROV) is a reemerging pathogen with no approved countermeasures, and it poses a growing public health threat. In response, we have developed a virus-like particle-based mRNA vaccine that elicits potent and durable neutralizing antibodies against both historical and circulating OROV strains, alongside a robust Th1-biased cellular immune response. This study reports the design and development of a critically needed vaccine candidate and provides fundamental insights into OROV antigenicity, thus demonstrating the utility of the mRNA platform for rapid response to emerging viral threats.

## INTRODUCTION

**Fig 1:**
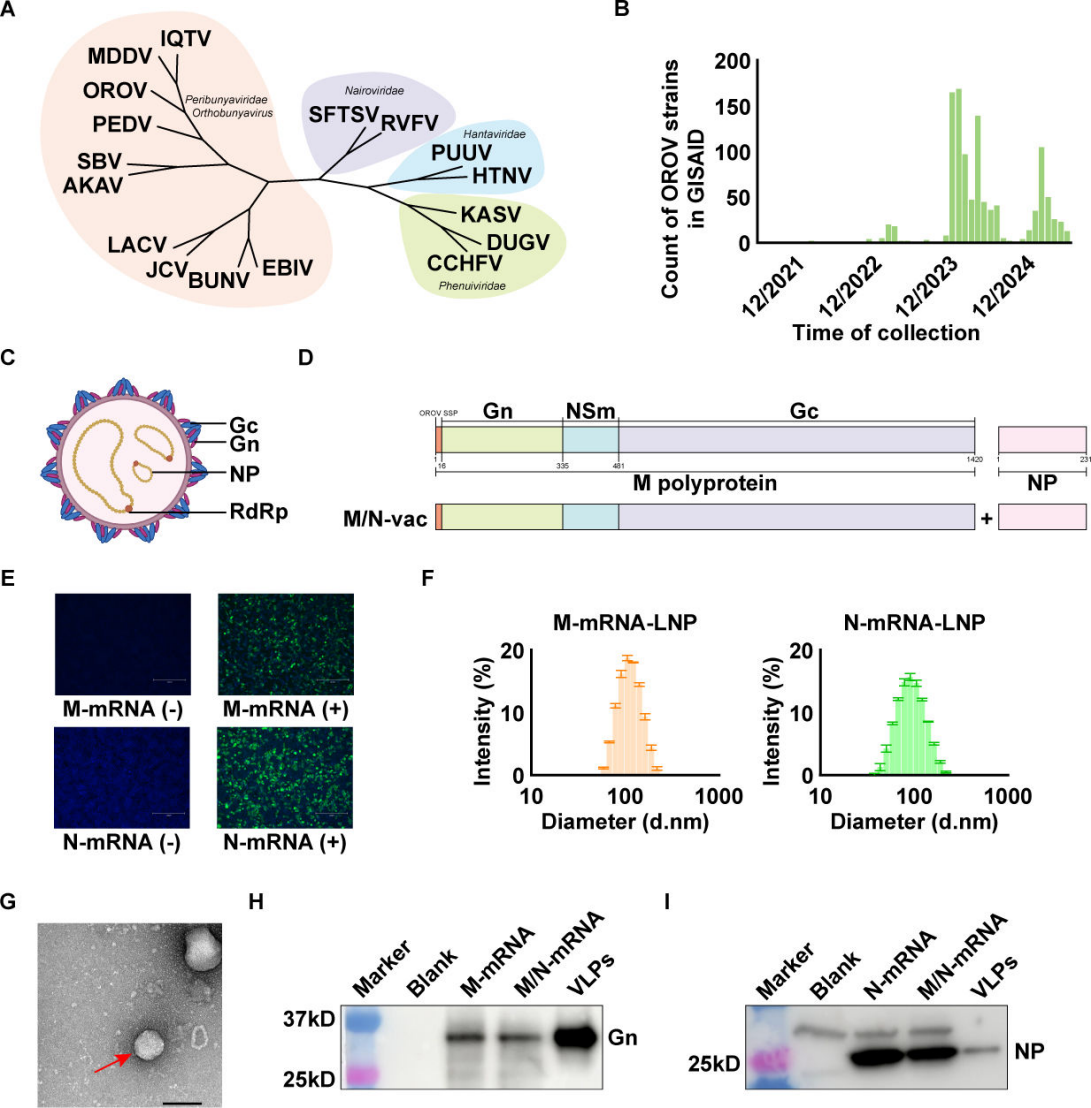
Construction of OROV mRNA vaccine. (**A**) Neighbor-joining phylogenetic tree analysis of the *Bunyaviricetes* class. (**B**) Number of OROV strains collected from June 2021 to June 2025 in the GISAID EpiArbo database. Data were obtained from GISAID on 26 July 2025. (**C**) Schematic diagram of OROV virion structure. Gc, Gc glycoprotein; Gn, Gn glycoprotein; NP, nucleocapsid protein; RdRp, RNA-dependent RNA polymerase. (**D**) Antigen design of OROV mRNA vaccine. Schematic illustration of open reading frames for the M polyprotein and NP of OROV (top). Antigen design of OROV M/N-vac mRNA vaccine candidate (bottom). OROV SSP, OROV secretion signal peptide. (**E**) Expression validation of transcribed mRNA in HEK293T cells, as detected by immunofluorescence assay. Scale bar = 300 μm. (**F**) Size distribution of mRNA-lipid nanoparticles (LNPs). Results are presented as the arithmetic mean ± standard deviation. (**G**) Transmission electron micrograph of OROV virus-like particles (VLPs). VLP is denoted with a red arrow. Scale bar = 100 nm. (**H**) Western blot analysis of Gn glycoprotein in lysates of mRNA-transfected HEK293T cells or purified OROV VLPs. (**I**) Western blot analysis of NP in lysates of mRNA-transfected HEK293T cells or purified OROV VLPs.

Oropouche virus (OROV), a member of the *Orthobunyavirus* genus (Fig. 1A) (https://ictv.global/taxonomy, accessed on 1 August 2025), is an arthropod-borne virus primarily transmitted by *Culicoides paraensis* (1). Since late 2023, novel OROV reassortants, such as hOROV/Brazil/AM-UKY-AM0088/2024, have triggered outbreaks across the Americas, and the geographic reach of OROV infection has rapidly expanded with approximately 30,000 confirmed cases to date (Fig. 1; Fig. S1A) (2, 3) (https://www.paho.org/en/arbo-portal/arbo-portal-oropouche, accessed on 28 July 2025). Notably, the first fatalities and vertical transmission events associated with OROV infection have now been reported (4, 5).

In addition to common febrile symptoms, severe neurological manifestations, including Guillain-Barré syndrome, as well as prolonged viremia, have been documented in several patients (6–8). Moreover, intercontinental travel-related cases highlight the growing risk of OROV spread beyond endemic regions, underscoring the potential threat of OROV to global public health (9). Therefore, this escalating and sustained public health burden calls for the development of effective interventions to prevent OROV infection and control outbreaks, particularly in vulnerable populations, such as pregnant women and newborns. Accordingly, elucidating OROV antigenicity and designing effective, durable, and broad-spectrum vaccines are critical priorities since no vaccines or therapeutics have been approved for clinical use against OROV, and fundamental aspects of its infection biology and antigenicity remain poorly understood.

OROV structural proteins include the glycoproteins and the nucleocapsid protein (NP) (Fig. 1C). The Gn and Gc glycoproteins are generated by proteolytic cleavage of the M polyprotein precursor and assemble into spike complexes on the viral envelope (Fig. 1D). These glycoproteins are considered essential for viral entry, positioning them as key targets for vaccine design and entry-inhibitor development (10). Nevertheless, the antigenic properties of OROV structural proteins remain largely uncharacterized, posing a major obstacle to the rational design of vaccines.

Owing to their rapid production, flexibility in antigen design, and capacity for co-delivery of multiple immunogens, mRNA vaccines represent a promising platform for combating emerging and reemerging infectious diseases like OROV (11, 12). Still, we do not know if mRNA vaccines can elicit more potent and durable immune responses compared to other vaccine modalities, such as virus-like particle (VLP)-based protein vaccines.

To systematically address this question, we herein report the design and development of an mRNA vaccine based on the prototype strain OROV/sloth/Brazil/PA-UG-BeAn19991/1960. The candidate vaccine, designated as M/N-vac, co-expresses the M polyprotein and NP, enabling self-assembly into OROV VLPs. In BALB/c mice, M/N-vac elicited robust and durable humoral and cellular immune responses, including high-titer neutralizing antibodies against pseudoviruses of the prototype strain and cross-neutralizing activity against the contemporary circulating strain hOROV/Brazil/AM-UKY-AM0088/2024. Furthermore, the vaccine induced a potent and sustained interferon-gamma (IFN-γ)-secreting Th1-biased cellular immune response directed against conserved epitopes within the nucleocapsid protein. These results demonstrate the immunogenicity and cross-reactive potential of a broad-spectrum OROV mRNA vaccine candidate. When compared to VLP-based protein vaccine, the M/N-vac mRNA candidate demonstrated superior immunogenicity in eliciting OROV-specific binding antibodies, cross-neutralizing antibodies, and cellular immunity, highlighting the advantages of the mRNA platform in responding to emerging viral threats. Collectively, our mechanistic study provides fundamental insights into OROV antigenicity, while our systematic approach aimed to design and develop a promising mRNA vaccine candidate for the prevention and control of OROV infection.

## RESULTS

### Design of OROV mRNA vaccines based on the antigens displayed in VLPs

Based on the OROV prototype strain OROV/sloth/Brazil/PA-UG-BeAn19991/1960 (Fig. 1D) and our study of the antigenic characteristics of OROV, we designed M/N-vac, an mRNA vaccine that co-delivers M polyprotein and NP as antigen. The expression of antigens was validated *in vitro* in HEK293T cells (Fig. 1E). The mRNAs were encapsulated with cationic lipid nanoparticles (LNPs) to form mRNA-LNP complexes with measured particle sizes around 100 nm (Fig. 1F).

Previous studies have suggested that co-expression of viral structural proteins can lead to the formation of VLPs (13). Here, we co-expressed the M polyprotein and the NP in a eukaryotic expression system in order to characterize the antigenic forms of co-delivery of the mRNA encoding both proteins. Following purification via ultracentrifugation, OROV VLPs were obtained. Under electron microscopy, these particles exhibited a diameter of approximately 100 nm, consistent with the size of native OROV virions (Fig. 1G). We then used Western blot (WB) to confirm the presence of both glycoproteins and NP in the purified VLP preparations (Fig. 1H and I). Since co-expression of M polyprotein and NP generates OROV VLPs, M/N-vac is confirmed to be an mRNA vaccine encoding antigens that form OROV VLPs.

### VLP-encoded mRNA vaccine elicited potent and durable antibodies against OROV infection

**Fig 2:**
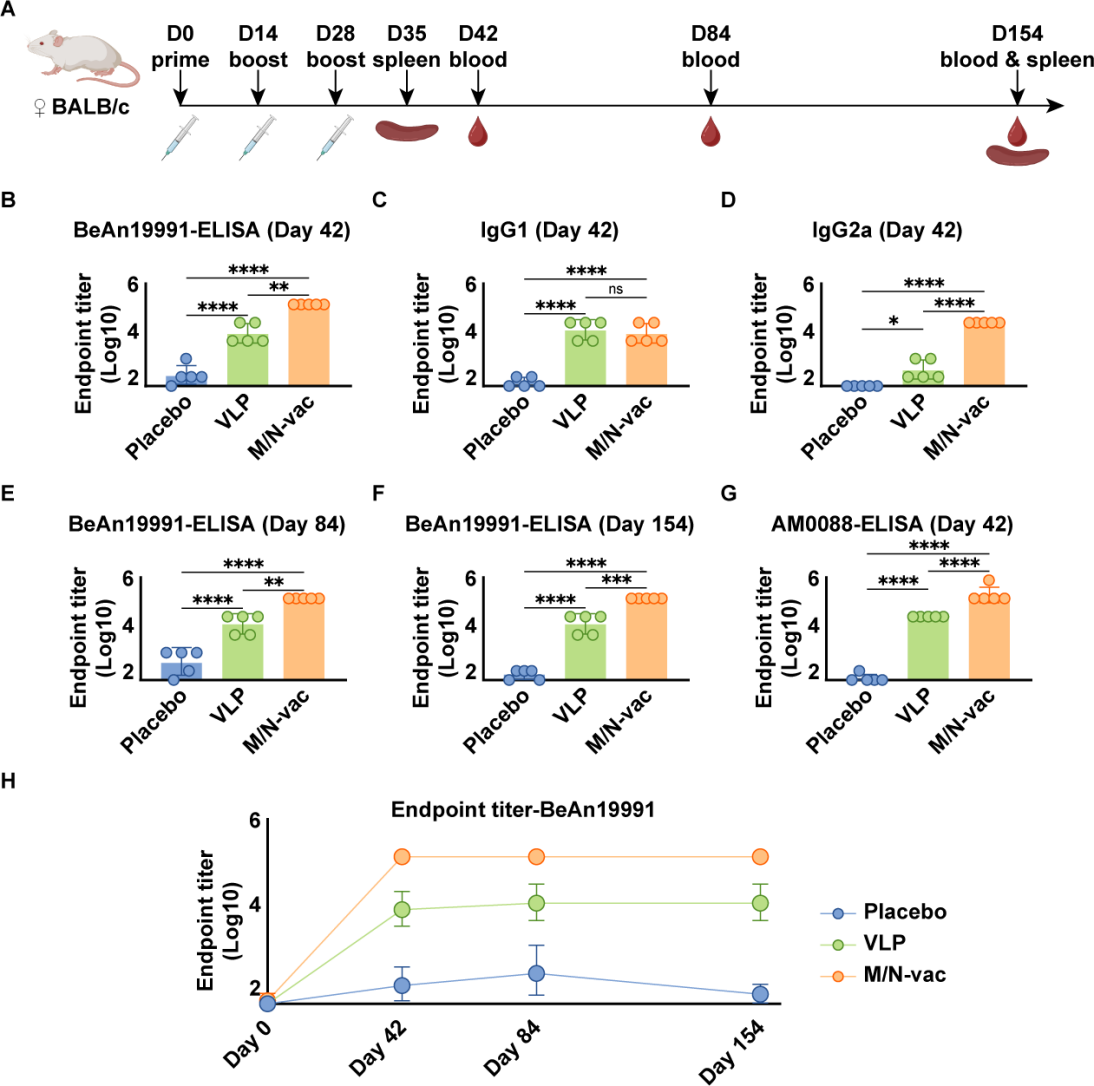
OROV VLP-specific antibody titers in sera from vaccine-immunized BALB/c mice. (**A**) Schematic of immunization procedure. (**B**) OROV VLP-specific IgG antibody endpoint titers in sera from immunized mice at 42 days post-prime immunization. (**C**) OROV VLP-specific IgG1 subclass antibody endpoint titers in sera from immunized mice at 42 days post-prime immunization. (**D**) OROV VLP-specific IgG2a subclass antibody endpoint titers in sera from immunized mice at 42 days post-prime immunization. (**E**) OROV VLP-specific IgG antibody endpoint titers in sera from immunized mice at 84 days post-prime immunization. (**F**) OROV VLP-specific IgG antibody endpoint titers in sera from immunized mice at 154 days post-prime immunization. (**G**) Endpoint titers of binding antibodies to VLPs of the contemporary circulating strain hOROV/Brazil/AM-UKY-AM0088/2024 in sera from immunized mice at 42 days post-prime immunization. (**H**) Kinetics of OROV VLP-specific IgG antibody endpoint titers in mice sera at 0, 42, 84, and 154 days post-prime immunization. The results in panels **B–H** are presented as geometric mean ± geometric standard deviation (*n* = 5). ns, not significant, *P < 0.05, **P < 0.01, ***P < 0.001, ****P < 0.0001*.*

To test the ability of M/N-vac to induce OROV VLP-specific immune responses, we immunized BALB/c mice with the M/N-vac mRNA vaccine. In parallel, we also immunized mice with OROV VLP-based protein vaccine. Sera were collected on days 42, 84, and 154 post-prime immunization (Fig. 2A), and the geometric mean titer (GMT) of OROV VLP-specific IgG antibodies was measured by VLP-based enzyme-linked immunosorbent assay (ELISA). After the three-dose immunization regimen, the GMT against OROV/sloth/Brazil/PA-UG-BeAn19991/1960 VLPs of the vaccine groups at 42 days post-prime immunization was measured. The M/N-vac mRNA vaccine induced a high IgG antibody titer with a GMT of 129,600, approximately 18-fold higher than that of the VLP-based protein vaccine as a control (Fig. 2B).

To further delineate the humoral immune response elicited by the vaccines, we quantified the endpoint titer of OROV VLP-specific IgG1 and IgG2a subclasses in sera from immunized mice at 42 days post-prime immunization. For IgG1, the GMTs induced by the VLP vaccine and the M/N-vac mRNA vaccine were 10,549 and 7,372, respectively (Fig. 2C). For IgG2a, GMTs were 205 and 21,600, respectively (Fig. 2D). At 84 days post-prime immunization, total OROV VLP-specific IgG antibody titers remained stable in both groups (Fig. 2E and H). At 154 days post-prime immunization, OROV VLP-specific IgG antibody titers of the M/N-vac group remained high at 129,600, while the VLP protein vaccine maintained a GMT of 10,549 (Fig. 2F and H).

To further evaluate the cross-reactive IgG antibodies of the vaccines, we measured antibody titers in mouse sera collected at 42 days post-prime immunization against the VLPs of the contemporary circulating strain hOROV/Brazil/AM-UKY-AM0088/2024. The M/N-vac mRNA vaccine performed better with a GMT of 185,454, approximately ninefold higher than that of the VLP vaccine (Fig. 2G).

### The VLP-encoded mRNA vaccine elicited potent neutralizing antibodies against pseudoviruses of homologous OROV strain BeAn19991 and heterologous strain AM0088

**Fig 3:**
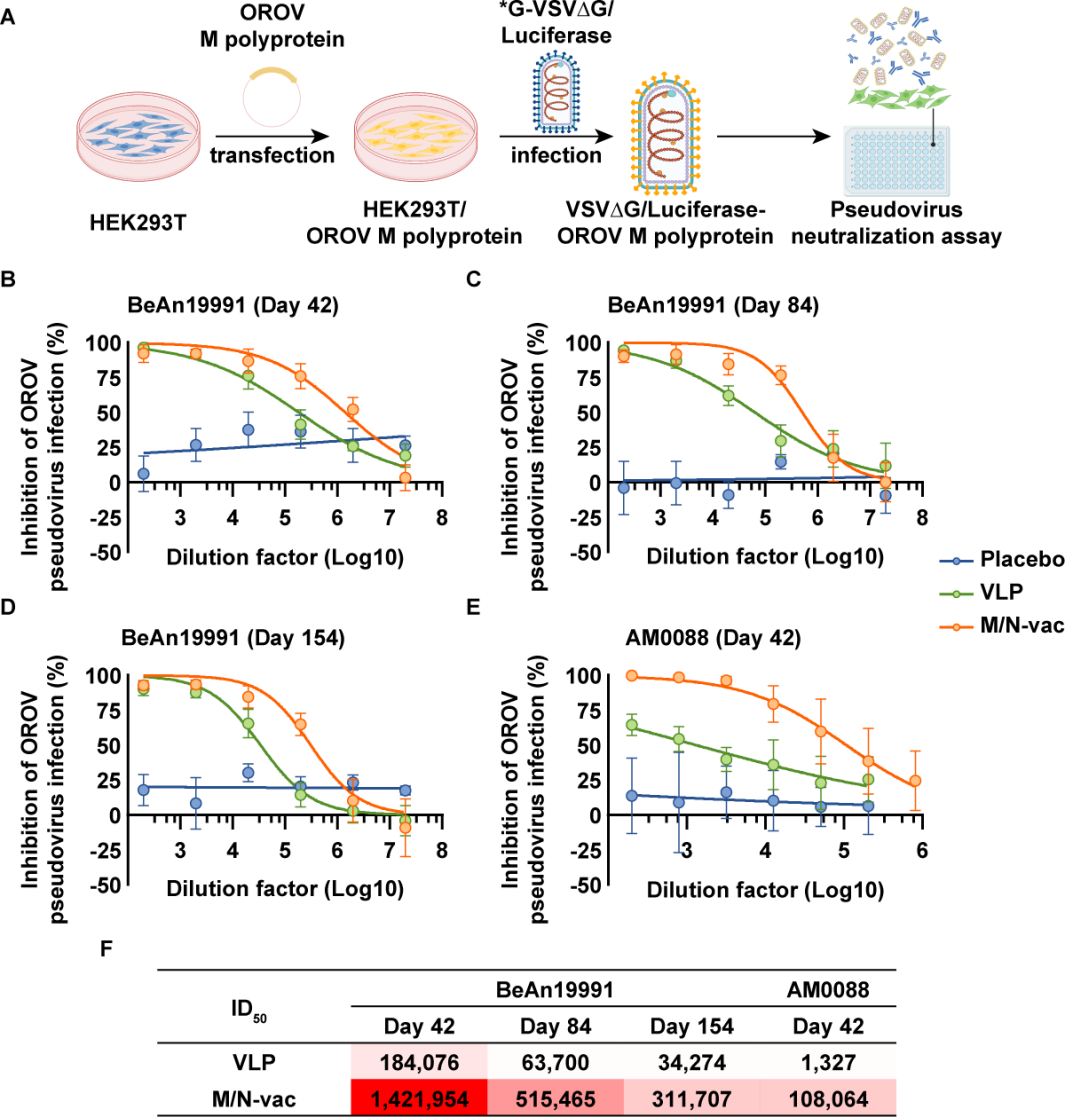
M/N-vac mRNA vaccine elicited potent and durable neutralizing antibody responses against OROV pseudovirus. (**A**) Schematic diagram of OROV pseudovirus production and neutralization assay. (**B**) Neutralization curves against the OROV prototype strain OROV/sloth/Brazil/PA-UG-BeAn19991/1960 pseudovirus of mouse sera at 42 days post-prime immunization. (**C**) Neutralization curves against the OROV prototype strain OROV/sloth/Brazil/PA-UG-BeAn19991/1960 pseudovirus of mouse sera at 84 days post-prime immunization. (**D**) Neutralization curves against the OROV prototype strain OROV/sloth/Brazil/PA-UG-BeAn19991/1960 pseudovirus of mouse sera at 154 days post-prime immunization. (**E**) Neutralization curves against the contemporary circulating strain hOROV/Brazil/AM-UKY-AM0088/2024 pseudovirus of mouse sera at 42 days post-prime immunization. (**F**) Half-maximal inhibitory dilution (ID_50_) values from the OROV pseudovirus neutralization assay. The results in panels** B–E** are presented as arithmetic mean ± standard deviation.

Neutralizing antibodies are crucial for preventing and controlling pathogenic infections, and they serve as a key correlate of protection in vaccine efficacy evaluation (14–17). To compare the capacity of the M/N-vac mRNA vaccine and VLP protein vaccine to elicit such antibodies, we measured OROV-specific neutralizing titers using a pseudovirus system. (Fig. 3A). The vesicular stomatitis virus (VSV)-based OROV pseudovirus could infect cell lines known to be susceptible to live OROV virus, such as Huh-7 (18, 19), Vero (20, 21), and Neuro-2a (22) cells (Fig. S2A). In this study, we used the Huh-7 cell line to demonstrate the specificity of the OROV pseudovirus system (Fig. S2B and C) and to determine neutralizing antibody titers.

We found that both vaccines designed on the basis of the OROV/sloth/Brazil/PA-UG-BeAn19991/1960 sequence induced neutralizing antibodies against the pseudovirus of the prototype (i.e., homologous) strain OROV/sloth/Brazil/PA-UG-BeAn19991/1960. At 42 days post-prime immunization, the half-maximal inhibitory dilution (ID_50_) values of sera from mice immunized with M/N-vac and VLP vaccines against OROV/sloth/Brazil/PA-UG-BeAn19991/1960 pseudovirus were 1.4 × 10^6^ and 1.8 × 10^5^, respectively (Fig. 3B). At 84 days post-prime immunization, these values were 5.2 × 10^5^ and 6.3 × 10^4^, respectively (Fig. 3C). At 154 days post-prime immunization, the ID_50_ values were 3.1 × 10^5^ and 3.4 × 10^4^, respectively (Fig. 3D). Next, we asked if both vaccines could elicit cross-neutralizing responses against the contemporary circulating (i.e., heterologous) strain hOROV/Brazil/AM-UKY-AM0088/2024. Accordingly, we tested sera collected at 42 days post-prime immunization for neutralization of hOROV/Brazil/AM-UKY-AM0088/2024 pseudovirus. The ID_50_ values of M/N-vac and VLP vaccines were 1.1 × 10^5^ and 1.3 × 10^3^, respectively (Fig. 3E and F), indicating that the VLP-encoded mRNA vaccine M/N-vac elicits more potent neutralizing antibodies than the VLP protein vaccine against both homologous strain OROV/sloth/Brazil/PA-UG-BeAn19991/1960 and heterologous strain hOROV/Brazil/AM-UKY-AM0088/2024.

### The VLP-encoded mRNA vaccine elicited a robust Th1-biased OROV VLP-specific cellular immune response

**Fig 4:**
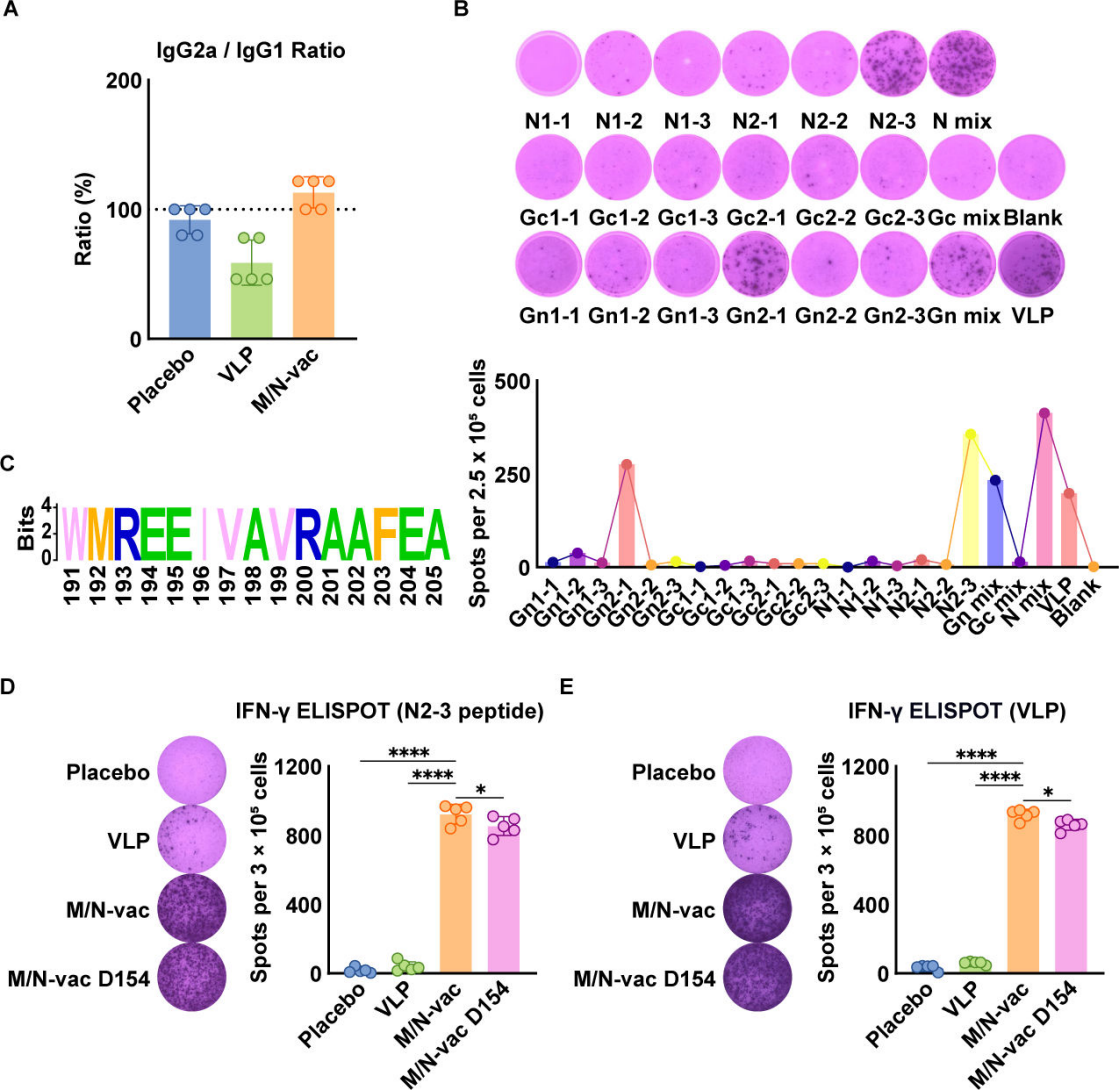
M/N-vac mRNA vaccine elicited potent and durable IFN-γ-secreting cellular immune responses in mice. (**A**) IgG2a/IgG1 endpoint titer ratio in sera of immunized mice at 42 days post-prime immunization. The results are presented as geometric mean ± geometric standard deviation. (**B**) Induction of IFN-γ-secreting splenocytes from M/N-vac-immunized mice by predicted epitope peptides. (**C**) Similarity logo plot of the N2-3 epitope in the NP from 1,095 OROV strains. (**D**) Number of epitope peptide N2-3-induced IFN-γ-secreting splenocytes at 35 days post-prime immunization. (**E**) Number of VLP-induced IFN-γ-secreting splenocytes at 35 days post-prime immunization. The results in panels** A**, **D**, and **E** are presented as arithmetic mean ± standard deviation (*n* = 5). ns*,* not significant, *P < 0.05, **P < 0.01, ***P < 0.001, ****P < 0.0001*.*

To further evaluate the cellular immunity elicited by the vaccines, we quantified the IgG2a/IgG1 ratios. Compared to the VLP protein vaccine, the M/N-vac mRNA vaccine showed a higher ratio at more than 1, suggesting a Th1-biased immune response (Fig. 4A) (23, 24).

We further investigated the induction of cellular immunity using enzyme-linked immunospot (ELISPOT) assay to quantify the number of IFN-γ-secreting splenocytes in response to stimulation with epitope peptides or VLPs at 35 days post-prime immunization. More specifically, to determine if NP could induce Th1-biased cellular immune response, we first predicted MHC-I/II epitopes using the Immune Epitope Database (IEDB) in BALB/c mice (Table 1). Then, using the ELISPOT assay, we found that the MHC-II epitope N2-3 within the OROV NP induced the strongest IFN-γ-secreting responses (Fig. 4B). Furthermore, sequence similarity analysis of 1,095 strains showed that peptide N2-3 is a potential immunodominant epitope and that it is highly conserved across OROV variants (Fig. 4C). ELISPOT assays further demonstrated that the M/N-vac mRNA vaccine elicited a higher IFN-γ-secreting cell response upon N2-3 peptide or VLP stimulation than the VLP protein vaccine (Fig. 4D and E).

**TABLE 1:** Predicted epitope peptides of OROV

MHC	Name	Amino acid sequence
MHC-I	Gn1-1	MPYSMIEAM
	Gn1-2	TYMPYSMI
	Gn1-3	KPALEATTKF
	Gc1-1	VYKKIGSEV
	Gc1-2	TYQELHNCI
	Gc1-3	AYRADLNT
	N1-1	VPQRTTSTF
	N1-2	RSPIKQAEF
	N1-3	RPMVDLTF
MHC-II	Gn2-1	PVTWFYGKVYKKINS
	Gn2-2	FSCTEALKVHRMGKD
	Gn2-3	TTVFSKNKPALEATT
	Gc2-1	IETYYKSNAAAYRAD
	Gc2-2	THHYKPTKNLPHVVP
	Gc2-3	GGKYYYSDSKEHAKD
	N2-1	MSEFIFNDVPQRTTS
	N2-2	PEAAYVAFEARYGQV
	N2-3	WMREEIVAVRAAFEA

Next, to investigate the longevity of cellular immune response, we isolated splenocytes from M/N-vac-vaccinated mice at 154 days post-prime immunization and stimulated with either the N2-3 peptide or VLPs to assess the IFN-γ-secreting Th1-biased cellular immune response. Although a slight decrease in IFN-γ secretion was observed compared to day 35, the M/N-vac mRNA vaccine still elicited a robust and sustained response at this late time point compared to the VLP protein (Fig. 4D and E), indicating that the M/N-vac mRNA vaccine is more effective than the VLP protein vaccine in eliciting long-term IFN-γ-secreting Th1-biased cellular immune response.

## DISCUSSION

OROV is an emerging and reemerging pathogen currently causing severe outbreaks in the Americas. However, because of long-term neglect, research on OROV epidemiology and molecular virology remains limited. No specific vaccines or therapeutics have been approved to date, and fundamental questions remain to be elucidated. For example, it is unclear which immunogen confers optimal immunogenicity and which vaccine platform most effectively induces protective immunity. Given the regional and global public health threat posed by OROV, it is imperative to develop effective, durable, and broad-spectrum vaccines and to systematically investigate OROV antigenicity.

In this study, we designed an mRNA vaccine co-expressing the M polyprotein and nucleocapsid protein, demonstrating that their co-expression leads to the assembly of OROV VLPs. Using a VLP-based ELISA, we evaluated the immunogenicity of vaccines presenting VLPs. Both the M/N-vac mRNA vaccine and the VLP protein vaccine induced high titers of OROV VLP-specific IgG antibodies, indicating strong immunogenicity of the VLP structure (Fig. 2B, E and F). Notably, the capacity to elicit pseudovirus-neutralizing antibodies varied substantially between the vaccine candidates. The M/N-vac mRNA vaccine induced significantly higher neutralizing activity against both the prototype strain and currently circulating variants compared to the VLP vaccine (Fig. 3F).

As a segmented negative-sense RNA virus, OROV has a high potential for mutation and reassortment, underscoring the importance of developing broad-spectrum vaccines. Although serological studies of natural infection suggest only limited cross-neutralization, the presence of cross-reactive antibodies supports the feasibility of a pan-OROV vaccine (2, 25). In our study, the M/N-vac mRNA vaccine based on the prototype strain OROV/sloth/Brazil/PA-UG-BeAn19991/1960 elicited a strong cross-neutralizing antibody response against the contemporary strain hOROV/Brazil/AM-UKY-AM0088/2024, about 80-fold higher than that induced by the VLP protein vaccine (Fig. 3E and F). While previous studies have identified multiple reactive linear epitopes on OROV glycoproteins (26), our findings suggest that the nucleocapsid protein also contributes substantially to broad immune protection; that is, co-expression of the nucleocapsid protein and M polyprotein elicited high-titer IgG antibodies reactive to VLPs from both prototype and circulating strains (Fig. 2B and G). Moreover, we identified a highly conserved potential immunodominant epitope in BALB/c, N2-3, within the nucleocapsid protein, highlighting its potential to elicit a broad-spectrum Th1-biased cellular immune response. Through rational antigen selection and sequence optimization (27–30), a safe and effective pan-OROV vaccine could be developed to establish herd immunity and protect vulnerable groups, such as pregnant women and young children.

IgG antibody titers in both vaccine groups remained elevated at 154 days post-immunization. Although neutralizing antibody titers in the M/N-vac group declined compared to earlier time points, they were sustained at relatively high levels and retained strong pseudovirus-neutralizing activity (Fig. 3F). Furthermore, the M/N-vac mRNA vaccine elicited durable antigen-specific cellular immune responses (Fig. 4D and E).

Several OROV vaccine candidates have been reported, including replication-deficient infectious clones (31), VSV-OROV chimeric viruses (26), recombinant Gc-spike protein (32), and epitope-based peptide vaccines (33, 34). However, these studies have largely emphasized technical and methodological developments, while fundamental aspects of OROV vaccinology and antigenicity remain underexplored. Moreover, a systematic evaluation of vaccine-induced OROV-specific humoral and cellular immunity has been lacking.

To address these deficiencies in this work, we developed non-replicating OROV vaccines based on mRNA and VLP platforms and established a suite of experimental models to systematically assess OROV-specific immune responses. The M/N-vac mRNA vaccine elicited robust and durable humoral immunity in BALB/c mice, including high-titer binding and neutralizing antibodies with cross-neutralizing activity against the contemporary circulating strain hOROV/Brazil/AM-UKY-AM0088/2024. Furthermore, it induced a potent and sustained Th1-biased cellular immune response. Notably, the VLP-encoded mRNA vaccine outperformed the protein-based VLP vaccine in eliciting both neutralizing antibodies and cellular immunity. This study not only provides a promising vaccine candidate for controlling OROV outbreaks but also constitutes a substantive advance in the understanding of OROV antigenicity and vaccine design.

It is important to acknowledge the limitations of this study. First, owing to biosafety constraints, limited access to authentic virus, and an incomplete understanding of OROV transmission routes and pathogenicity, we were unable to evaluate serum neutralization efficacy against live virus, choosing, instead, a pseudovirus approach. Second, all vaccine candidates were designed based on the prototype strain OROV/sloth/Brazil/PA-UG-BeAn19991/1960 to systematically evaluate optimal antigen formats and delivery strategies for OROV vaccination; circulating strains have not been incorporated into the vaccine design at this stage.

## MATERIALS AND METHODS

### Cell lines

HEK293T cells, Huh-7 cells, Vero cells, Vero E6 cells, BHK-21 cells, U-87 MG cells, HeLa cells, and Neuro-2a cells were obtained from the American Type Culture Collection. Cells were cultured in Dulbecco’s Modified Eagle Medium (MeilunBio) supplemented with 10% fetal bovine serum and 1% penicillin-streptomycin-amphotericin B at 37°C and 5% CO_2_. Expi-293F cells were cultured in 293F Hi-Expression Medium (Shanghai OPM Biosciences) at 37°C and 8% CO_2_, with shaking at 115 rpm.

### Peptides

All peptides were synthesized with ≥90% purity and verified by high-performance liquid chromatography (Synpeptide).

### Plasmids

The codon-optimized nucleocapsid protein (GenBank: AJE24680.1) and M polyprotein (GenBank: AJE24679.1) of OROV prototype strain OROV/sloth/Brazil/PA-UG-BeAn19991/1960 were synthesized and cloned into the pcDNA3.1(+) vector (GenScript). A FLAG tag (DYKDDDDK) was added to the C-terminus of the nucleocapsid protein, and an HA tag (YPYDVPDYA) was added between the OROV signal peptide (residue M1-G16) and the N-terminus of the Gn glycoprotein. The codon-optimized M polyprotein (GenBank: XHP15871.1) of the OROV contemporary circulating strain hOROV/Brazil/AM-UKY-AM0088/2024 (2, 3) was synthesized and cloned into the pcDNA3.4 vector (GenScript).

The mRNA vaccine template plasmids were constructed based on the amino acid sequence of the OROV prototype strain OROV/sloth/Brazil/PA-UG-BeAn19991/1960. The insert of the M mRNA plasmid is the M polyprotein fused to an N-terminus HA tag, as described previously. The insert of the N mRNA plasmid is the nucleocapsid protein fused to the C-terminus FLAG tag, as described previously.

### *In vitro* transcription and LNP encapsulation

The mRNA vaccine plasmids were linearized and then used as the DNA template for *in vitro* transcription as previously described (35). mRNA was transcribed using the HiScribe T7 High Yield RNA Synthesis Kit (NEB) with N1-Methyl-Pseudo-UTP (NEB). Cap-1 mRNA was synthesized with Vaccinia Capping Enzyme and mRNA Cap 2′-O-methyltransferase (NEB). mRNA was purified with the Monarch Spin RNA Cleanup Kit (NEB). mRNA was encapsulated using NanoAssemblr  Ignite (Cytiva). The particle size of mRNA-LNP was measured by dynamic light scattering using Malvern Zetasizer Nano-ZS (Malvern) as previously described (35). The encapsulation efficiency of mRNA-LNP was measured by the Quant-iT RiboGreen RNA Reagent and Kit (Invitrogen).

### Immunofluorescence assay

The expression of mRNA was verified through the immunofluorescence assay *in vitro*. Briefly, HEK293T cells were transfected with 3 µg of capped mRNA using Lipofectamine 3000 (Invitrogen). After 48 h, the cells were fixed with 4% paraformaldehyde and 0.05% Triton X-100. After blocking with 5% non-fat powdered milk, the cells were incubated with the primary antibody for 90 min at 37°C: rabbit anti-DYKDDDDK-tag recombinant antibody (1:500, 80801-2-RR; Proteintech) for FLAG tag detection and rabbit anti-HA-tag polyclonal antibody (1:500, 51064-2-AP; Proteintech) for HA tag detection. After washing with PBST four times, the cells were incubated with the secondary antibody for 45 min at 37°C: goat anti-rabbit IgG H&L Alexa Fluor 488 (1:1,000; ab150077; Abcam). After washing with PBST four times again, the cells were stained with DAPI. Fluorescence images were captured using the EVOS M5000 Imaging System (Invitrogen).

### WB

The protein composition of OROV VLPs was determined through Western blot, as previously described (36). HEK293T cells were transfected with 3 µg of capped mRNA using Lipofectamine 3000 (Invitrogen) as a control. After 48 h, the cells were washed with PBS and lysed in 1× RIPA buffer. Protein concentration of the cell lysate was measured with the BCA Protein Assay Kit (Takara). Five micrograms of cell lysate or VLPs was subjected to 10% SDS-PAGE gel separation. After transfer to a PVDF membrane and blocking in TBST containing 5% BSA at 25°C for 1 h, the membrane was incubated with primary antibody at 4°C overnight: rabbit anti-DYKDDDDK-tag recombinant antibody (Proteintech; 1:10,000; 80801-2-RR) for FLAG tag detection and rabbit anti-HA-tag polyclonal antibody (Proteintech; 1:10,000; 51064-2-AP) for HA tag detection. Then, the membrane was incubated with HRP-conjugated secondary antibody for 1 h at room temperature. The PVDF membrane was washed using TBST after each antibody incubation. The chemiluminescent signal was excited via SuperSignal West Pico PLUS (Thermo Fisher Scientific), and the images were captured using ChemiDoc (Bio-Rad).

### Expression and purification of virus-like particles

Briefly, for the expression of OROV OROV/sloth/Brazil/PA-UG-BeAn19991/1960 VLPs, the expression plasmids of M polyprotein and nucleocapsid protein were co-transfected into Expi-293F cells using EZ *Trans* (Life iLAB). The supernatants were collected after 5 days. Following the removal of cells and cellular debris, the VLPs were further purified by ultracentrifugation. Ultracentrifugation was performed in Optima L-100 XP (BECKMAN) using an SW 32 Ti rotor at 30,000 rpm for 2 h at 4°C with a 5 mL cushion of 20% (wt/vol) sucrose in TNE buffer underlaid in the tube. Subsequently, the pellet was resuspended in TNE buffer, and VLP protein concentration was quantified using the BCA Protein Assay Kit (Takara). VLPs were stored at −80°C until use.

### Animal vaccination

Specific-pathogen-free (SPF) female BALB/c mice (8 weeks old) were divided into three groups (*n* = 5 per group), designated Placebo, M/N-vac, and VLP. Animals were housed in the SPF barrier facility at the Laboratory Animal Center of Fudan University. All groups received intramuscular immunizations of the same vaccine type and dose on Days 0, 14, and 28 of the immunization schedule: Group Placebo received 100 μL PBS buffer per mouse; Group M/N-vac was co-immunized with 5 μg M mRNA-LNP and 5 μg N mRNA-LNP per mouse; Group VLP received 5 μg OROV OROV/sloth/Brazil/PA-UG-BeAn19991/1960 VLPs formulated with aluminum adjuvant at a 1:1 (vol/vol) ratio per mouse. Serum samples were collected on Days 42, 84, and 154 post-prime immunization, and splenocytes were harvested on Day 154. Concomitantly, a parallel cohort of mice, identically grouped and immunized, was used exclusively for splenocyte collection on Day 35 post-prime immunization.

### ELISA

ELISA was used to quantify the OROV VLP-specific IgG, IgG1, and IgG2a titers in sera as described previously (36). Briefly, wells of a 96-well ELISA microplate (Corning) were coated with 1 µg/mL OROV VLPs overnight at 4°C. The coated wells were blocked for 2 h at 37°C. Serum samples of mice were diluted serially, starting at a dilution of 1:100. This was followed by incubation in the ELISA-coated microplate for 1 h at 37°C. After washing five times with PBST, HRP-conjugated secondary antibody was applied to the wells and incubated for 30 min at 37°C. HRP-conjugated goat anti-mouse IgG (1:10,000; Dako), HRP-conjugated goat anti-mouse IgG1 (1:5,000; Thermo Fisher Scientific), and HRP-conjugated goat anti-mouse IgG2a (1:2,000; Thermo Fisher Scientific) were used to detect OROV VLP-specific IgG, IgG1, and IgG2a in mice. After incubation with the secondary antibody, the wells were washed five times with PBST, followed by adding 3,3′,5,5′-tetramethylbenzidine to visualize the reaction. Finally, H_2_SO_4_ was used to stop the reaction, and the absorbance at 450 nm was measured using the EnSight multi-mode microplate reader (PerkinElmer). The ELISA endpoint titer was defined as the highest serum dilution at which the absorbance was measured at 450 nm. Once the titers were below 1:100, they were defined as 1:40. The endpoint ELISA titer for each group was reported as the GMT calculated from the individual endpoint titers of all mice within that group (*n* = 5).

### Production of the vesicular stomatitis virus-based OROV pseudovirus

VSV-based single-round-infection OROV pseudoviruses were generated as previously described (37, 38). Briefly, HEK293T cells were transfected with plasmids encoding the M polyprotein of OROV strains OROV/sloth/Brazil/PA-UG-BeAn19991/1960 or hOROV/Brazil/AM-UKY-AM0088/2024. After 24 h, the transfected cells were infected for 2 h at 37°C with *G-VSVΔG/Luciferase, a pseudovirus in which the VSV-G gene was replaced by the Renilla luciferase gene. Then, the cells were washed with PBS three times and replenished with DMEM with 10% FBS containing 1 μg/mL anti-VSV-G monoclonal antibody I1. After incubation for 24 h, the supernatant containing the OROV pseudovirus VSVΔG/Luciferase-OROV M glycoprotein was harvested and stored at −80°C.

### Pseudovirus neutralization assay

The pseudovirus neutralization assay was performed as previously described (35, 39, 40). Briefly, Huh-7 cells were seeded in wells of a 96-well plate at a density of 10,000 cells per well and incubated for 24 h before the assay. Mouse sera were inactivated at 56°C for 30 min. Sera were serially diluted in DMEM. An equal volume of pseudovirus was added to the diluted sera, and the mixture was incubated at 37°C for 30 min. Following the removal of the cell culture medium, the sera-pseudovirus mixture was added to the cells. After 12 h incubation at 37°C, the supernatant was replaced with fresh DMEM containing 4% FBS. Cells were cultured for an additional 36 h, after which the supernatant was discarded, and the cells were lysed. Renilla luciferase activity was quantified through the Renilla Luciferase Assay System (Promega) using the EnSight multi-mode microplate reader (PerkinElmer). The luminescence (relative light units, RLU) of virus control wells (pseudovirus only) was defined as the reference value. Neutralizing sera could reduce luminescence relative to the reference value. The serum dilution factor that reduced luminescence to 50% of the reference value was defined as the ID_50_. ID_50_ was calculated using nonlinear regression analysis of the neutralization curve.

### ELISPOT assay

The ELISPOT assay was performed using Mouse IFN-γ ELISpot Flex (ALP) (MabTech) according to the manufacturer’s instructions as previously described (36). Splenocytes were isolated from immunized mice and prepared as single-cell suspensions. Splenocytes were seeded in wells of a Multiscreen hydrophobic PVDF-membrane 96-well plate (Merck) at a density of 300,000 cells per well and stimulated with 2 μg/well OROV OROV/sloth/Brazil/PA-UG-BeAn19991/1960 VLPs or 1 μg/well predicted epitope peptides for 48 h. Spots were developed using the substrate of ALP and counted with ImmunoSpot Analyzers (Cellular Technology Ltd.).

### Prediction of the epitope peptides of OROV

MHC class I and II epitopes within the Gn, Gc, and nucleocapsid proteins of OROV prototype strain OROV/sloth/Brazil/PA-UG-BeAn19991/1960 were predicted for BALB/c mice using the IEDB (https://www.iedb.org/) (41). The top three highest-scoring peptides were selected as candidate epitopes with the amino acid sequences listed in Table 1.

### Epidemiological analysis

The epidemiological findings of this study are based on data obtained from the GISAID EpiArbo database (42) on 26 July 2025 or the Pan American Health Organization (PAHO) on 28 July 2025.

### Phylogenetic analysis

We conducted a phylogenetic analysis of the *Bunyaviricetes* class using amino acid sequences of the M polyprotein from some typical viruses belonging to the *Bunyaviricetes* class. M polyprotein sequences were aligned using MAFFT (version 7) (43), and a neighbor-joining phylogenetic analysis (bootstrap = 500) was conducted and visualized with MEGA 12 (version 12.0.11) (44). The phylogenetic tree was modified in iTOL (version 7.2.1, https://itol.embl.de/) (45). Sequences obtained from the NCBI were listed as follows: Hantaan virus (NP_941978.1), Puumala virus (NP_941983.1), Bunyamwera virus (NP_047212.1), La Crosse virus (YP_010839410.1), OROV (AJE24679.1), Iquitos virus (XOL54509.1), Madre de Dios virus (AHY22342.1), Perdões virus (AJT39462.1), Schmallenberg virus (YP_009666912.1), Ebinur Lake virus (YP_010840764.1), Jamestown Canyon virus (YP_009666885.1), Akabane virus (YP_001497160.1), Crimean-Congo hemorrhagic fever virus (NP_950235.1), Dugbe virus (NP_690575.1), Kasokero virus (YP_009246488.1), Rift Valley fever virus (YP_003848705.1), and Severe fever with thrombocytopenia syndrome virus (YP_009666134.1).

### Epitope similarity analysis

Amino acid sequences of the nucleocapsid protein of 1,095 OROV strains were obtained from the GISAID EpiArbo database. The sequences of the N2-3 epitope in nucleocapsid protein (residues 191–205) were aligned using MAFFT (version 7) and visualized using WebLogo (https://weblogo.berkeley.edu/, version 2.8.2) (46).

### Quantification and statistical analysis

All statistical analyses were performed using GraphPad Prism 9. One-way ANOVA with Tukey’s multiple comparison tests was used to evaluate statistical significance. Results with *P* < 0.05 were considered statistically significant (*P < 0.05, **P < 0.01, ***P < 0.001, ****P < 0.0001; ns, not significant).

## SUPPLEMENTAL MATERIAL

Supplemental Figures mbio.03653-25-s0001.docx Fig. S1 and S2.

ASM does not own the copyrights to Supplemental Material that may be linked to, or accessed through, an article. The authors have granted ASM a non-exclusive, world-wide license to publish the Supplemental Material files. Please contact the corresponding author directly for reuse.
