# Supplementary material for: A VLP-based mRNA vaccine elicits potent humoral and cellular immunity against Oropouche virus
Source: mBio. 2026 Jan 12;17(2):e03653-25. doi: 10.1128/mbio.03653-25 (PMC12892965; doi:10.1128/mbio.03653-25)
Supplement: Supplemental Figures — Fig. S1 and S2. [file mbio.03653-25-s0001.docx]

**Supplemental material**

**A VLP-based mRNA vaccine elicits potent humoral and cellular immunity against Oropouche virus**

**Yuren Shi,^1^ Guangxu Zhang,^1^ Siyu Lin,^2^ Mengyu Hu,^1^ Yuanzhou Wang,^1^ Haoyu Ge,^1^ Shuai Xia,^1^ Qian Wang,^1,*^ Shibo Jiang,^1,*^ Lu Lu^1,*^**

^1^Key Laboratory of Medical Molecular Virology (Ministry of Education/National Health Commission/Chinese Academy of Medical Science), Shanghai Institute of Infectious Disease and Biosecurity, School of Basic Medical Sciences, Shanghai Frontiers Science Center of Pathogenic Microbes and Infection, Shanghai Public Health Clinical Center, Fudan University, Shanghai, 200032, China

^2^School of Basic Medicine, Dali University, Dali, 671000, China

^*^Address correspondence to Qian Wang ([wang_qian@fudan.edu.cn](mailto:wang_qian@fudan.edu.cn)), Shibo Jiang ([shibojiang@fudan.edu.cn](mailto:shibojiang@fudan.edu.cn)), Lu Lu ([lul@fudan.edu.cn](mailto:lul@fudan.edu.cn)).

Yuren Shi and Guangxu Zhang contributed equally to this work. Author order was

determined by drawing straws.

**Keywords:** Oropouche virus (OROV), mRNA vaccine, virus-like particles (VLPs), cross-reactive antibodies


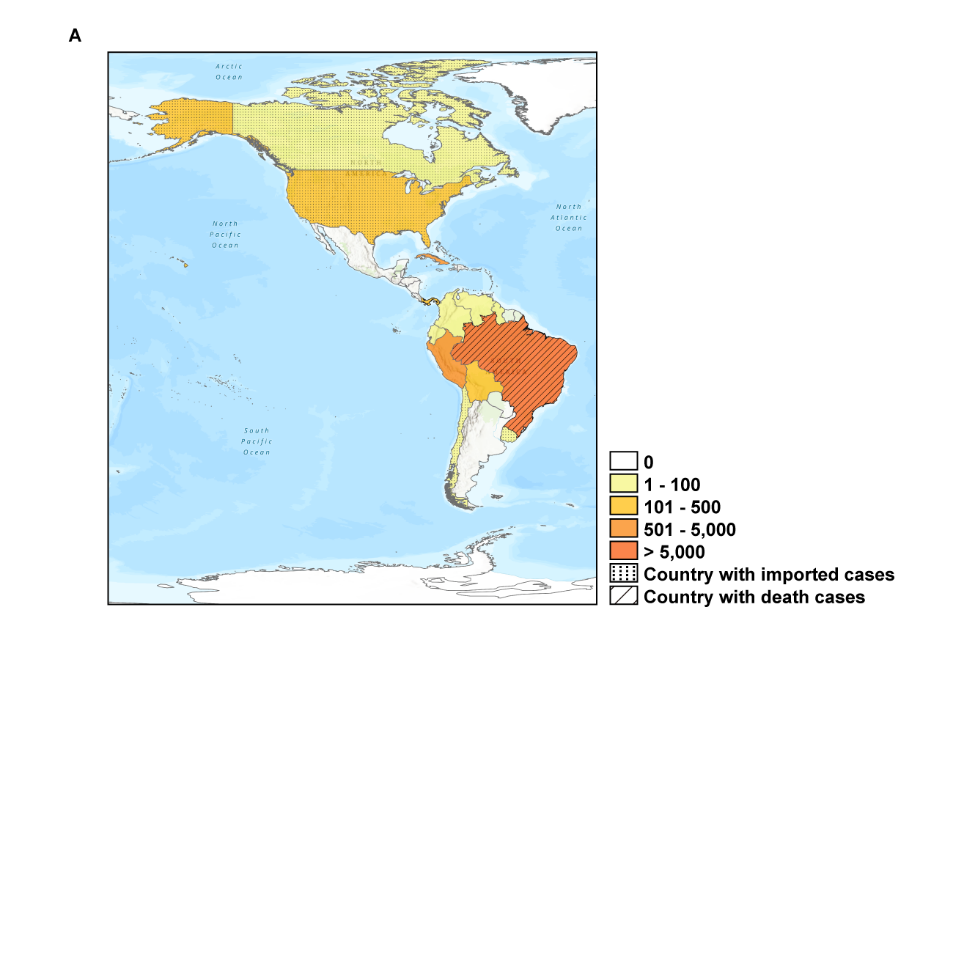


**Figure S1.** Spatial distribution of Oropouche virus (OROV) reports. (A) Geographical distribution of confirmed OROV cases, deaths, and imported cases in the Americas. Data were obtained from PAHO on July 28, 2025 (<https://www.paho.org/en/arbo-portal/arbo-portal-oropouche>).


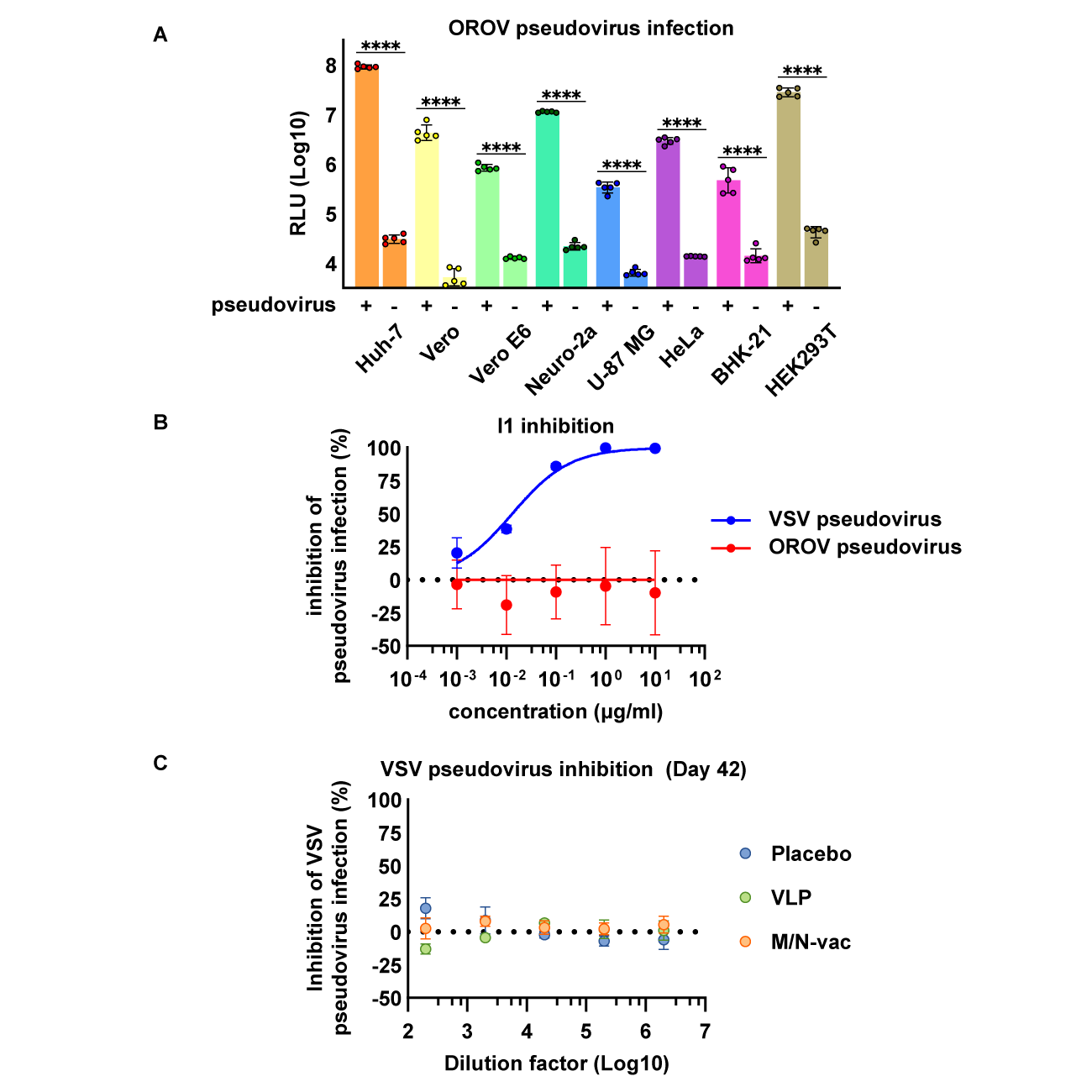


**Figure S2.** Infectivity and specificity of OROV pseudovirus. (A) Luminescence (relative light units, RLU) of OROV prototype strain OROV/sloth/Brazil/PA-UG-BeAn19991/1960 pseudovirus infection in multiple cell types. Results are presented as the arithmetic mean ± standard deviation (AM ± SD) (n = 5). (B) The inhibition of the VSV pseudovirus and OROV prototype strain OROV/sloth/Brazil/PA-UG-BeAn19991/1960 pseudovirus by VSV-G neutralizing antibody I1 (produced by the I1 hybridoma; ATCC: CRL-2700). (C) Neutralization curves against the VSV pseudovirus of mouse sera at 42 days post-prime immunization. Results are presented as the AM ± SD (n = 5).
